# The AB Blood Group System Phenotype Does Not Play a Role in *Toxoplasma gondii* Infection in Cats

**DOI:** 10.3390/pathogens14121227

**Published:** 2025-12-01

**Authors:** Eva Spada, Greta Tattarletti, Daniela Proverbio, Roberta Perego, Luciana Baggiani, Giulia Donato, Rosalia D’Agostino, Francesca Arcuri, Paola Galluzzo, Giuseppina Chiarenza, Valeria Blanda, Francesca Grippi

**Affiliations:** 1 Department of Veterinary Medicine and Animal Sciences, University of Milan, 96900 Lodi, Italy; greta.tattarletti@studenti.unimi.it (G.T.); roberta.perego@unimi.it (R.P.); luciana.baggiani@unimi.it (L.B.); 2 Department of Veterinary Sciences, University of Messina, 98122 Messina, Italy; giudonato@unime.it; 3 Istituto Zooprofilattico Sperimentale della Sicilia “A. Mirri”, 90129 Palermo, Italy; rosalia.dagostino@izssicilia.it (R.D.); francesca.arcuri@izssicilia.it (F.A.); paola.galluzzo@izssicilia.it (P.G.); giuseppina.chiarenza@izssicilia.it (G.C.); valeria.blanda@izssicilia.it (V.B.); francesca.grippi@izssicilia.it (F.G.)

**Keywords:** *Toxoplasma gondii*, feline blood phenotypes, AB blood group system, risk factors, seroprevalence

## Abstract

Previous studies have evaluated the association between different blood groups and human infection with *Toxoplasma gondii*. No similar studies exist in cats. The objective of this study was to evaluate the role of some risk or protective factors, including the AB blood type system phenotypes, in *T. gondii* infection in cats. Feline sera and surplus EDTA anticoagulated blood samples, for which AB blood group system phenotypes had been determined, were analyzed for *T. gondii* antibodies (ELISA, cut-off S/P% ≥ 50% and IFAT, cut-off ≥ 1:64) and DNA (nested and real-time PCR). *T. gondii* status and the characteristics of signalment (gender, breed, and age), lifestyle (stray, shelter, privately-owned), origin (Northern or Southern Italy), and retroviral infection serostatus of the population were evaluated using the Chi-square test, with calculation of the Odds Ratio (OR) in cases of statistically significant association (*p* < 0.05). A total of 199 samples were analyzed, of which 178 were phenotype A, 15 were phenotype B, and 6 were phenotype AB. Of these, 57/199 (28.6%) were positive for *T. gondii*: 5 were positive at PCR, 33 at ELISA, and 19 at IFAT. Of the 57 positive cats, 52/57 were phenotype A, 3/57 phenotype B, and 2/57 phenotype AB, with no significant association with *T. gondii* infection. FIV seropositive cats had a higher risk (OR = 3.1, *p* = 0.0043) of testing *T. gondii* positive. This study did not find an association between *T. gondii* infection and the feline blood types investigated; therefore, based on our results, AB blood group system phenotypes do not seem to play a role in *Toxoplasma gondii* infection in cats. These findings contribute to our knowledge of the role of blood types in disease susceptibility in cats.

## 1. Introduction

Blood group antigens are molecular structures found on the surface of erythrocytes and other tissues, composed mainly of polysaccharides, glycoproteins, and glycolipids. These molecules, which vary among individuals of the same species, exhibit antigenic properties that define different blood types. In humans, these antigens are known to influence susceptibility to several infectious diseases by acting as receptors for bacteria, viruses, and protozoa, thereby affecting host colonization and immune response [1]. For instance, certain blood group phenotypes are associated with increased resistance to malaria in endemic regions, likely due to evolutionary selection pressures [2].

The potential link between blood groups and *T. gondii* infection has been explored in human medicine, given the parasite’s worldwide distribution and zoonotic impact. *T. gondii* is an obligate intracellular protozoan parasite belonging to the phylum Apicomplexa. The parasite has a complex heteroxenous life cycle, with sexual development occurring in the intestines of definitive hosts (felids) and asexual replication taking place in extra-intestinal tissues of intermediate hosts, including humans. It is essential to acknowledge that cats and people play distinct roles in the life cycle of *T. gondii* infection. Cats produce oocysts, resulting in a short and predominantly intestinal infection [3]. In contrast, people are intermediate hosts who develop systemic and long-lasting infections, with dissemination of tachyzoites followed by persistence of tissue cysts. These biological differences lead to distinct patterns of exposure, immune responses, and host–parasite interactions [4,5]. Transmission primarily occurs through ingestion of oocysts excreted by infected felids [6,7]. As *T. gondii* uses the gastrointestinal tract as a route for infection, and ABO, Lewis, and Secretor histo-blood group carbohydrates are expressed in this organ [8,9], a potential biological relationship between them has been proposed. Some human studies have reported a higher susceptibility to *T. gondii* in individuals with blood groups B or AB [10,11], while others found associations with groups A and O [12]. Contradictory results exist, and a recent meta-analysis has shown no significant correlation between either ABO or Rh blood groups and infection [13,14,15,16,17]. These discrepancies highlight that well-known factors like eating contaminated undercooked meat, poultry, or shellfish; consuming unpasteurized goat’s milk; drinking contaminated water; and contact with cat feces or contaminated soil play a more significant role in the infection of *T. gondii* than blood group antigens [18,19].

In contrast to the extensive literature in human medicine, less is known about the role of blood types in the pathogenesis of infectious diseases in veterinary medicine. In cats, the primary blood group system is the AB system, which includes three blood phenotypes: A, B, and AB [20]. These blood phenotypes are defined by the expression of specific sialic acids—NeuGc for type A and NeuAc for type B—on red blood cells, regulated by the CMAH enzyme. AB cats express both NeuGc and NeuAc antigens in equal amounts on their erythrocyte surfaces [21]. Importantly, the feline antigens on the surface of erythrocytes are not restricted to these cells. The feline A blood group antigen is also found on feline lymphocytes [22], but to the author’s knowledge, no studies have investigated if these blood type antigens are also expressed in the gastrointestinal tract, as reported in human studies. In addition, blood group-related naturally occurring antibodies could interact with the parasite, influencing host susceptibility or resistance, as demonstrated in SARS-CoV-2 infection both in people and in cats [23]. In humans, naturally occurring anti-A or anti-B antibodies in individuals with blood types O, B, and A can bind the SARS-CoV-2 S protein and block its interaction with ACE2 receptors, thus preventing viral attachment and entry [24,25]. These antibodies may also opsonize viral particles and promote complement-mediated neutralization [26], contributing to the protection of individuals with blood group O during the SARS outbreak. In contrast, type AB people lack both anti-A and anti-B antibodies and therefore lack the protective effect of these antibodies. Similarly, cats with the rare AB blood phenotype have no naturally occurring alloantibodies [27], which may explain their higher risk for SARS-CoV-2 seropositivity in a recent study [28].

Whilst no studies have yet evaluated the relationship between feline blood types and *T. gondii* infection, as discussed previously for human medicine, feline blood group antigens may influence susceptibility to the parasite. This study aimed to explore the potential association between feline blood phenotypes A, B, or AB and *T. gondii* infection. In addition, potential risk and protective factors were evaluated, including origin, lifestyle, signalment, and retroviral coinfections that might influence this infection in cats.

## 2. Materials and Methods

This observational study was conducted on stray, shelter, and owned cats from different provinces in Lombardy (northern Italy) and Sicily (southern Italy). Blood samples and animal data were collected between 2012 and 2022 during routine health check-ups, before neutering, or as part of preventative health assessments. Blood typing was performed on anticoagulated fresh blood or on refrigerated stored blood within 7 days from blood collection, while *T. gondii* analyzes were performed on stored frozen blood or serum. Only surplus serum or EDTA anticoagulated whole blood samples with informed owner consent were used for this study. Based on the University of Milan’s animal use regulations, formal ethical approval was therefore not needed as cats were sampled for diagnostic purposes and informed consent was given by the owners, the director of the shelters, and the legal representatives of the feline colonies for surplus blood samples and data to be used for scientific purposes.

### 2.1. Population

A total of 199 samples were available for this study. For most cats, the following data were recorded: signalment (breed, sex, reproductive status, age [kittens < 1 year; adults 1–10 years; seniors > 10 years] [29], lifestyle (owned, shelter or stray), origin (northern vs. southern Italy) and serostatus for retroviral infections (feline immunodeficiency virus, FIV and/or feline leukemia virus, FeLV) using a commercial SNAP COMBO PLUS^®^ rapid enzyme-linked immunosorbent assay (ELISA) kit (IDEXX Laboratories Srl, Milan, Italy) that detects, in serum, plasma or whole blood samples, FIV-specific anti-p24 and anti-gp40 antibodies and FeLV antigen p27.

### 2.2. Blood Typing

AB blood group system phenotyping was performed at the Veterinary Transfusion Research Laboratory (REVLab), Department of Veterinary Medicine and Animal Sciences (DIVAS), University of Milan, Italy, using the standard tube agglutination [20]. All phenotype B and AB samples were confirmed through immunochromatographic (LabTEST A+B, Alvedia, Limonest, France) and back-typing methods as previously described [30].

### 2.3. Molecular Test for the Identification of T. gondii Genome

DNA of *T. gondii* was extracted from the EDTA whole-blood samples and analyzed using both nested PCR and real-time PCR at the Istituto Zooprofilattico Sperimentale (IZS) of Sicily, Italy. Nested PCR was carried out using the primers Toxo P1 5′-GGAACTGCATCCGTTCATGAG-3′, Toxo P2 5′-TGCATAGGTTGCAGTCACTG-3′, Toxo P3 5′-GGCGACCAATCTGCGAATACACC-3′, Toxo P45′-TCTTTAAAGCGTTCGTGGTC-3′, targeting a fragment of the *B1* gene. The amplification reactions were performed in a SimpliAmp Thermal Cycler (Applied biosystems by Thermo Fisher Scientific (Waltham, MA USA), as previously described [31].

The primers used for the real time PCR were as follows: forward 5′-CACAGAAGGGACAGAAGT-3′, reverse ‘5-TCGCCTTCATCTACAGTC-3′, and the TaqMan probe 5′-FAM-CTCTCCTCCAAGACGGCTGG-BHQ1, amplifying a region of the 529-bp repeat element [32]. Reactions were carried out in a QuantStudio 6 Flex (Applied biosystems by Thermo Fisher Scientific (Waltham, MA USA).

### 2.4. Serological Test for the Detection of T. gondii Antibodies Using the ELISA Method

Detection of anti-*T. gondii* IgG antibodies was performed on serum samples at the Istituto Zooprofilattico Sperimentale della Sicilia using a commercial indirect ELISA kit for multiple species, including cats (ID Screen^®^ Toxoplasmosis Indirect Multi-species, ID.Vet—Innovative Diagnostics SAS, Grabels, France). The test is based on the detection of antibodies directed against the P30 antigen of *T. gondii.* For each sample, the S/P% was calculated as follows: (OD sample − OD negative control)/(OD positive control − OD negative control) × 100, with serum samples presenting S/P% ≥ 50% considered positive, between 40% and 50% as doubtful and ≤40% as negative. The samples were read using a Multiskan Skyhigh spectrophotometer (Thermo Fisher Scientific, Waltham, MA, USA) set at 450 nm. The results were interpreted as a dichotomous outcome—seropositive or seronegative.

### 2.5. Serological Test for the Detection of T. gondii Antibodies Using the IFAT Method

Serum samples were tested for anti-*T. gondii* IgG using the Fluo *T. gondii* CAT kit (Biopronix, Agrolabo S.p.A., Scarmagno, Torino, Italy) at the Veterinary Transfusion Research Laboratory (REVLab), Department of Veterinary Medicine and Animal Sciences (DIVAS), University of Milan, Italy. Samples were screened at 1:32 dilution and, if positive, serially diluted up to 1:128 in PBS. The cutoff for seropositivity was set at ≥1:64. Slides pre-coated with *T. gondii* antigens were incubated with serum, washed, and treated with FITC-labeled anti-feline IgG conjugate. Fluorescence was evaluated at 400× magnification using a ZEISS Axioskope microscope (Carl Zeiss AG, Oberkochen, Germany). Positive samples showed bright green fluorescence outlining tachyzoites.

### 2.6. Statistical Analyzes

The data were analyzed using standard descriptive statistics and reported as mean ± standard deviation (SD) or median and range, depending on their distribution. Univariate analysis of categorical data using Chi-squared test was performed to determine possible associations between *T. gondii* infection and the different variables considered. Associations were considered statistically significant with a probability (*p*) value < 0.05. Odds ratios (OR) were used to assess any association between the AB blood group phenotypes, other risk factors, and *T. gondii* infection. Cats were considered *T. gondii* infected if they tested seropositive for *T. gondii* antibodies and/or PCR positive for *T. gondii* DNA. In case of multiple factors significantly associated with the infective status, a multivariate analysis via logistic regression with the stepwise method was performed. Statistical analysis was performed using a commercially available software (MedCalc Statistical Software version 23.0.6, Ostend, Belgium; 2019).

## 3. Results

Demographic data and feline retroviruses serostatus of surveyed cats are summarized in Table 1. Exact age was available for 40 cats and resulted in a median age of 2 years (range: 4 months–14 years, 25–75% percentile: 1 year–6 years).

Of 199 serum samples, 77 were tested by IFAT, with 19 positives, while 122 were tested by ELISA, with 33 positives. Therefore, taking together the results of serological analyses, 52 samples were seropositive for *T. gondii* antibodies. Five samples were PCR positive, for a total of 57/199 infected cats, yielding a prevalence of infection of 28.6%. The results of univariate analysis (Chi-squared test) among factors and status of infection for *T. gondii* are reported in Table 1. Being FIV-positive was significantly associated with *T. gondii* infection according to the univariate analysis (OR = 3.1; 95%CI = 1.4–6.7, *p* = 0.0043).

Blood phenotype A was identified in 178 cats (89.4%), phenotype B in 15 cats (7.5%), and phenotype AB in 6 cats (3%). All phenotype B and AB samples were confirmed by immunochromatographic and back-typing techniques. Out of 57 *T. gondii* positive samples, 52 were blood phenotype A, 3 were blood phenotype B, and 2 were phenotype AB, with no statistically significant association with infective status (Table 2).

## 4. Discussion

The primary aim of our study was to explore the potential association between feline blood groups and *T. gondii* infection. Our research results confirmed the null hypothesis that no association exists between Toxoplasma infection and blood groups in cats. Our results, in fact, did not show any significant association between feline AB blood group types and *T. gondii* infection, suggesting that, in contrast to results in human studies, antigenic expression is unlikely to have a meaningful impact on host susceptibility. One possible explanation for this result is that the role of cats as definitive hosts is different from that of intermediate hosts. In people, blood group antigens have been hypothesized to influence susceptibility to *T. gondii* due to the systemic nature of infection [3,4,5]. In cats, the infection is primarily localized to the intestinal epithelium, which may limit the impact of antigenic variation on susceptibility.

Increasing evidence suggests that blood group antigens play a significant role in host susceptibility to various infections across species. These antigens may function as receptors or coreceptors for microorganisms, parasites, and viruses, influencing infection outcomes.

The ABO blood group system has been extensively studied in people in relation to malaria susceptibility. Research indicates that individuals with blood group O have a reduced risk of severe *Plasmodium falciparum* malaria compared to those with non-O blood groups. This mechanism may be explained by the absence of A and B antigens on O erythrocytes, which limits the ability of infected cells to form rosettes with uninfected ones, a process closely linked to severe malarial pathology, although the exact mechanisms remain under investigation [2,33,34]. In rabbits, histo-blood group antigens (HBGAs), are expressed on the surface of a variety of tissues such as the duodenum, trachea, and biliary ducts [35]. A well-documented association exists between histo-blood group antigens (HBGAs) and susceptibility to rabbit hemorrhagic disease virus (RHDV), where HBGA expression influences viral binding and infection outcomes [36]. This supports the broader hypothesis that blood group antigens may act as facilitators in the pathogenesis of infectious diseases across species. Another study in cats has investigated the relationship between blood groups and susceptibility to hemoplasma infections. While no direct preferential binding to blood phenotypes A or B antigens was consistently observed, a significant association has been reported between the Ab genotype (phenotypes A or AB) and an increased likelihood of hemoplasma infection [37]. The underlying mechanisms behind this association remain unclear, but it has been suggested that blood group antigens may influence host–pathogen interactions, either by modulating innate immune responses or by affecting pathogen recognition and clearance.

As a secondary objective of this study, we assessed whether factors such as signalment (sex, neuter status, age, breed), geographic origin (Northern vs. Southern Italy), lifestyle (owned, stray, or shelter cats), and other infections like retroviruses could represent potential risk factors for infection.

When we assessed whether *T. gondii* infection was associated with retroviral infection, FIV seropositive cats had a significantly higher risk (OR = 3.1, *p* = 0.0043) of testing *T. gondii* positive. Clinical cases of toxoplasmosis have been documented in cats infected with FIV or FeLV, suggesting that retrovirus-induced immunosuppression is a significant risk factor for the development of clinical disease [38,39]. FIV primarily infects CD4+ T-helper cells, B lymphocytes, and macrophages, leading to progressive immunodeficiency. This immunosuppression facilitates both primary infection and reactivation of latent *T. gondii* cysts, increasing the risk of clinical toxoplasmosis. Additionally, the administration of immunosuppressive agents, such as glucocorticoids or cyclosporine, has been associated with reactivation of latent *T. gondii* infections in immunocompromised cats [40,41]. These findings highlight the importance of monitoring retrovirus-positive cats for *T. gondii* infection and support the hypothesis that retroviral immunosuppression, particularly due to FIV, may facilitate both increased susceptibility to infection and reactivation of latent toxoplasmosis.

No significant associations were found between infection and variables such as age, sex, breed, lifestyle or reproductive status. Contrary to the findings of some previous studies, age was not identified as a risk factor in our population. Nevertheless, other research has reported a higher seroprevalence in adult and elderly cats, likely due to increased cumulative exposure over time [42,43]. Similarly, sex was not associated with infection, consistent with previous studies [44,45]. However, some authors have reported a higher prevalence in intact males, potentially due to increased roaming behavior and contact with contaminated environments [43,46]. No association was found between lifestyle and infection; however, previous investigations have reported that lifestyle is one of the main factors influencing the likelihood of acquiring *T. gondii* infection, with higher seroprevalence in stray and shelter cats compared with owned cats [47,48,49]. In Italy, stray colony cats are often fed by people but still have the freedom to hunt, placing them at a similar risk level as feral cats described in the literature [49]. Regarding breed, although some studies have suggested higher infection rates in mixed-breed or certain purebred cats [50], again, no significant association was observed in our data.

This study adds some new data on the prevalence of *T. gondii* infection in cats from Italy. Several studies have examined the seroprevalence and risk factors of *T. gondii* infection worldwide, with rates of seropositivity reaching up to 60% among stray and feral cat populations [51]. In some European populations, reported seropositivity rates range between 18.2% and 40.7% [44,52]. An Italian study reported a seropositivity rate of 30.5% in stray and shelter cats in Northern Italy [53], while another study in Tuscany found seroprevalence greater than 30% [54]. In our study, we found an overall seropositivity rate of 26.1%, a result consistent with previously cited regional data. Considering also the PCR results, the infection rate resulted in 28.2% in northern Italy and 28.0% in Southern Italy (specifically in Sicily). To the author’s knowledge, the data on feline toxoplasma infection prevalence in Sicily is original new data, as no information is available on this infection in this area.

Of the whole blood samples analyzed using PCR, only 5 yielded a positive result. Although PCR is highly specific and effective for detecting active parasitemia, its sensitivity is limited by the transient and low-level presence of *T. gondii* in peripheral blood, particularly in chronic or latent infections [55,56]. This observation is consistent with previous studies that have reported variable blood PCR positivity rates in feline populations. For instance, a study conducted in China reported a PCR positivity rate of 5.2% in stray cats [57], while another study found a positivity rate of 0.7% in cats from western Mexico [58]. Additionally, a study in South Korea reported a PCR-positive rate of 13.2% in stray cats [59]. These variations may be attributed to several factors, including geographical location, environmental conditions, dietary habits, and specific methodologies employed in each study.

A limitation of this study is the absence of clinical evaluation of toxoplasma-positive cats; disease severity and onset were not analyzed, with focus only on infection status. This approach allowed us to have a general overview of infection prevalence but provided no information about the effect of blood group on disease progression or the severity of toxoplasmosis. In humans, the Duffy blood group system has been proposed as a risk factor linked to ocular toxoplasmosis, and it is reported that individuals with the Fy (a+b+) phenotype have a six-fold higher likelihood of developing ocular symptoms than those with the Fy(a−b−) phenotype [60]. Conversely, a subsequent study by Ferreira et al. [61] did not confirm this association. More details on development of signs would be essential to better understand individual risk factors and the potential implications for clinical management of toxoplasmosis in cats. Another limitation is that we tested only the blood phenotypes of the feline AB blood group system. However, new feline blood types have been discovered in recent years, and future studies could explore the involvement of other erythrocyte antigen systems, such as the Mik system [62] and feline erythrocyte antigen system (FEA 1—FEA 2—FEA 3—FEA 4—FEA 5) [63], in susceptibility to infection. In addition, we did not investigate the genotype of each cat in our population, and as previously discussed, a previous study found an association between genotype, but not with AB blood group system phenotypes, and hemoplasma infection [37].

## 5. Conclusions

In conclusion, this study did not identify any significant association between feline AB blood group systems phenotypes and *T. gondii* positivity. This underlines that these feline blood types probably do not play a role in this infection in cats. Although evidence on this topic in veterinary medicine remains limited, our findings contribute to a better understanding of the complex interactions between blood group systems and infectious diseases in cats. Further studies are warranted to clarify the role of blood types in the pathogenesis of infections across animal species.

## Figures and Tables

**Table 1:** Characteristics of the feline population of 199 cats investigated for the association between AB blood group system phenotype and antibodies against *T. gondii* and the parasite’s genome, and analysis for factors associated with seropositivity/state of infection according to univariate analysis with chi-square test. In bold, statistically significant *p* < 0.05.

Parameter (*n* = Number of Cats for Which the Data Was Available)	Variable	Number (%)	State of Infection		*p*-Value (Chi-Squared Test)
			Infected	Uninfected	
Origin *n* = 199	Northern Italy Southern Italy	149 (74.9) 50 (25.1)	43 14	106 36	0.9077
Lifestyle *n* = 183	Stray Shelter Owned	137 (74.9) 32 (17.5) 14 (7.7)	45 7 2	92 25 12	0.0883 0.2986 0.1950
Breed *n* = 174	DSH Non DSH	172 (98.9) 2 (1.1)	48 1	124 1	0.4910
Sex *n* = 183	Male Female	96 (52.5) 87 (47.5)	25 30	71 57	0.2148
Reproductive status *n* = 183	Intact Neutered	152 (83.1) 31 (16.9)	44 11	108 20	0.4706
Age classes *n* = 173	Kitten (<1 yr) Adult (1–10 yrs) Senior (>10 yrs)	28 (16.2) 138 (79.8) 7 (4.0)	5 44 4	23 94 3	0.1101 0.4807 0.1215
FIV serostatus *n* = 190	Positive Negative	22 (11.6) 168 (88.4)	12 41	10 127	**0.0031**
FeLV serostatus *n* = 190	Positive Negative	7 (3.7) 183 (96.3)	4 49	3 134	0.0795
FIV/FeLV coinfection serostatus *n* = 190	Positive Negative	2 (1.1) 188 (98.9)	1 52	1 136	0.4846

DSH: domestic shorthair; FIV: feline immunodeficiency virus; FeLV: feline leukemia virus.

**Table 2:** Effect of feline blood phenotypes A, B and AB on *T. gondii* infection status in an Italian population of 199 cats tested for possible association between AB blood group system phenotype and *T. gondii* infection.

Blood Phenotype	Infected *n* (%) *n* = 57	Uninfected *n* (%) *n* = 142	*p*-Value (Chi-Squared Test)
A	52 (91.2)	126 (88.7)	0.6053
B	3 (5.3)	12 (8.5)	0.4424
AB	2 (3.5)	4 (2.8)	0.7969

## Data Availability

The data presented in this study are available upon request from the corresponding authors.

## Abbreviations

The following abbreviations are used in this manuscript:

**ACE2**	Angiotensin Converting Enzyme 2
**DSH**	Domestic Shorthair
**EDTA**	Ethylenediaminetetraacetic Acid
**ELISA**	Enzyme-linked Immunosorbent Assay
**FIV**	Feline Immunodeficiency Virus
**FeLV**	Feline Leukemia Virus
**HBGA**	Histo-Blood Group Antigens
**IFAT**	Indirect Fluorescent Antibody Test
**IgG**	Immunoglobulin G
**OR**	Odds Ratio
**PCR**	Polymerase Chain Reaction
**RHDW**	Rabbit Hemorrhagic Disease Virus
***T. gondii***	Toxoplasma gondii
